# MORC2B is essential for meiotic progression and fertility

**DOI:** 10.1371/journal.pgen.1007175

**Published:** 2018-01-12

**Authors:** Baolu Shi, Jiangyang Xue, Jian Zhou, Seth D. Kasowitz, Yuanwei Zhang, Guanxiang Liang, Yongjuan Guan, Qinghua Shi, Mingxi Liu, Jiahao Sha, Xiaoyan Huang, P. Jeremy Wang

**Affiliations:** 1 State Key Laboratory of Reproductive Medicine, Nanjing Medical University, Nanjing, China; 2 Department of Biomedical Sciences, University of Pennsylvania School of Veterinary Medicine, Philadelphia, Pennsylvania, United States of America; 3 Center for Reproduction and Genetics, The Affiliated Suzhou Hospital of Nanjing Medical University, Suzhou, Jiangsu, China; 4 Xinhua Hospital, Affiliated to Shanghai Jiao Tong University School of Medicine, Shanghai, China; 5 USTC-SJH Joint Center for Human Reproduction and Genetics, School of Life Sciences, University of Science and Technology of China, Hefei,Anhui, China; 6 Department of Microbiology, University of Pennsylvania Perelman School of Medicine, Philadelphia, Pennsylvania, United States of America; Cornell University, UNITED STATES

## Abstract

The microrchidia (MORC) family proteins are chromatin-remodelling factors and function in diverse biological processes such as DNA damage response and transposon silencing. Here, we report that mouse *Morc2b* encodes a functional germ cell-specific member of the MORC protein family. *Morc2b* arose specifically in the rodent lineage through retrotransposition of *Morc2a* during evolution. Inactivation of *Morc2b* leads to meiotic arrest and sterility in both sexes. *Morc2b*-deficient spermatocytes and oocytes exhibit failures in chromosomal synapsis, blockades in meiotic recombination, and increased apoptosis. Loss of MORC2B causes mis-regulated expression of meiosis-specific genes. Furthermore, we find that MORC2B interacts with MORC2A, its sequence paralogue. Our results demonstrate that *Morc2b*, a relatively recent gene, has evolved an essential role in meiosis and fertility.

## Author summary

In sexually reproducing organisms, meiosis, a process unique to germ cells, produces haploid gametes. Abnormalities in meiosis can lead to infertility, loss of pregnancy, or genetic diseases such as Down syndrome. The meiotic processes are tightly regulated by a large number of genes including many meiosis-specific ones. The majority of meiosis-specific factors are conserved, however, species-specific factors have evolved. Here we report functional studies of a rodent lineage–specific gene named *Morc2b*. *Morc2b* belongs to a family of chromatin-remodelling factors. *Morc2b* is specifically expressed in germ cells. Disruption of *Morc2b* causes meiotic arrest and infertility in both sexes. Notably, MORC2B regulates the expression of a number of meiosis-specific genes. Interestingly, MORC2B interacts with its sequence homologue MORC2A. These functional studies have uncovered a new protein complex in the regulation of key meiotic processes and suggested the presence of continued selection pressure for evolution of new meiosis-specific factors.

## Introduction

The microrchidia (MORC) protein family forms a conserved class of chromatin remodeling factors found in diverse species from *Arabidopsis* to human [1]. MORC proteins contain GHKL-type (Gyrase, Hsp90, histidine kinase, MutL) ATPase domain and PHD zinc finger domain, implying functions related to DNA metabolism and epigenetic regulation. *Arabidopsis* AtMORC1 and AtMORC6 repress transposable elements in a methylation-independent manner and are essential for heterochromatin formation and gene silencing [2]. In mammals, four different MORC proteins (MORC1-4) have been identified [1]. Human MORC2 recruits histone deacetylases to promoter regions, causing local histone H3 deacetylation and transcriptional repression [3, 4]. MORC2 also modulates chromatin relaxation in response to DNA damage [5, 6]. MORC3 binds to H3K4me3 (trimethylated histone H3 lysine 4) in vitro and localizes to H3K4me3-marked genomic sites [7]. Collectively, these studies reveal a conserved role for MORC proteins in the regulation of high-order chromatin organization.

Mutations in *MORC2* cause axonal Charcot-Marie-Tooth disease (CMT) in humans [8]. CMT is a neural disorder characterized by muscle weakness and atrophy, and changes in the sensation in the body periphery. In neuronal cells, MORC2 is recruited to heterochromatin by the HUSH (human silencing hub) complex to compact chromatin and thus is required for epigenetic silencing [9]. The HUSH complex mediates H3K9me3 deposition in heterochromatin by SETDB1 (H3K9 trimethyltransferase) to maintain transcriptional silencing [10]. In addition, MORC2 promotes breast cancer invasion/metastasis and gastric tumorigenesis [6]. These studies demonstrate the critical role of *MORC2* in human diseases.

Genetic requirements of *Morc1* and *Morc3* in mouse have been reported. Mouse *Morc1*, the founding member of the MORC family, is specifically expressed in the male germline and its ablation results in male sterility with meiotic arrest [11, 12]. *Morc1* deficiency is associated with de-silencing of transposable elements in the male germline [13]. *Morc3*^-/-^ mice die at birth or within one day after birth [14]. Study of mice heterozygous for a *Morc3* mutation reveals a role in bone homeostasis [15]. However, the physiological functions of *Morc2* and *Morc4* are not known.

In mouse, two paralogues of *Morc2* are present: *Morc2a* and *Morc2b*. Mouse *Morc2b* was reported to be a transcriptional target of PRDM9, a histone H3 trimethyltransferase required for meiotic progression and involved in speciation [16]. *PRDM9* is the only known mammalian speciation gene [17]. PRDM9 specifies sites of preferred meiotic recombination (i.e. hotspots) and drives recombination away from functional genomic elements such as gene promoter regions [18]. Following sequence-specific DNA binding through its array of zinc fingers, PRDM9 catalyzes trimethylation of H3K4 (H3K4me3) and H3K36 (H3K36me3) [19–23]. In *Prdm9*-deficient testes, *Morc2b* is not expressed and H3K4me3 at the *Morc2b* promoter is low, suggesting that PRDM9 normally induces *Morc2b* expression via H3K4me3 [16]. However, the function of *Morc2b* remains unknown. Here, we report that *Morc2b* is required for chromosomal synapsis and meiotic recombination in both sexes. Inactivation of *Morc2b* causes mis-expression of a number of genes including meiosis-specific genes. We find that MORC2B interacts with MORC2A. The *Morc2b*-null mouse mutant exhibits meiotic defects similar to the *Prdm9*-null mutant, suggesting that MORC2B may be a key downstream effector of PRDM9 in meiosis.

## Results

### *Morc2b* is a retrotransposed homologue of *Morc2a*

**Fig 1:**
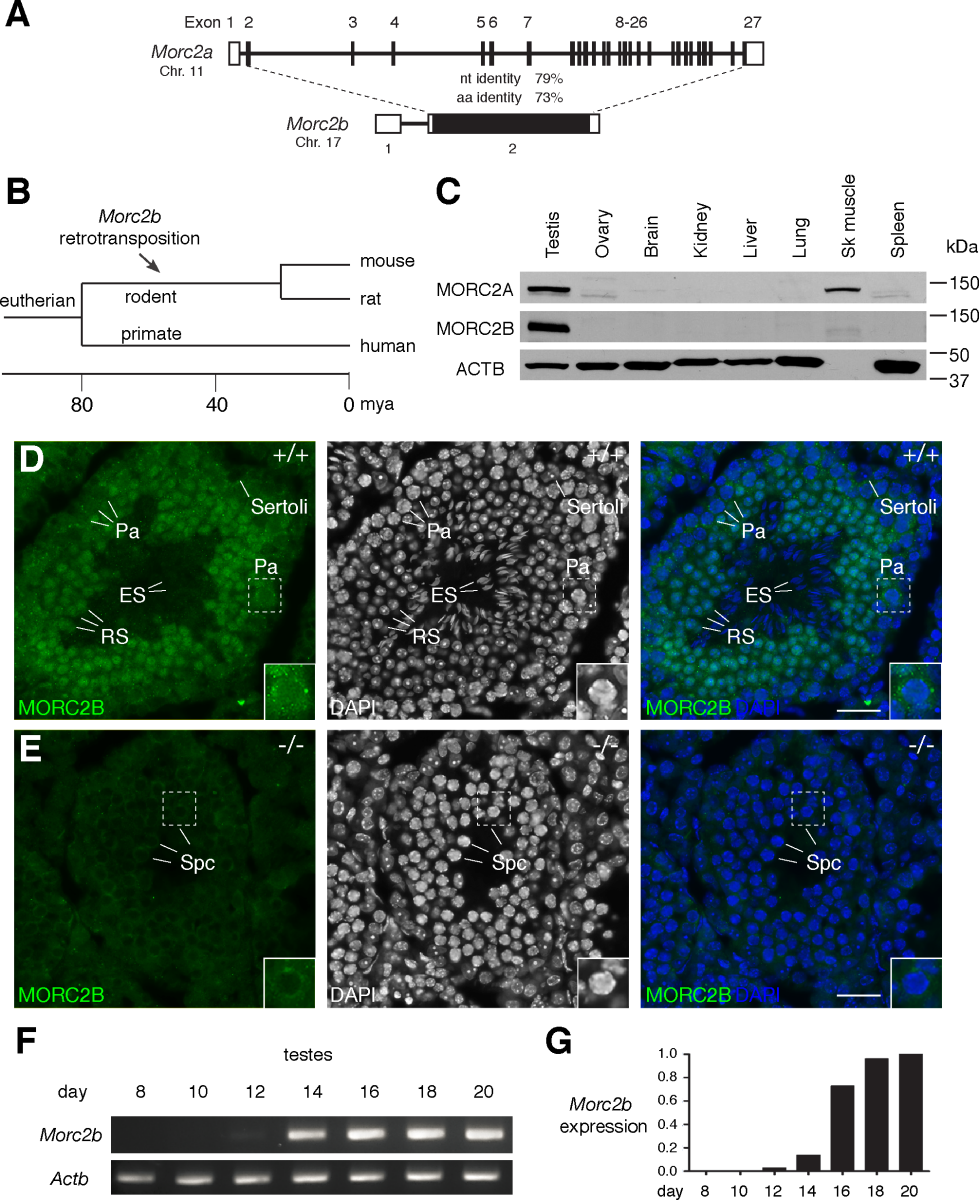
*Morc2b* is a retrotransposed germ cell-specific derivative of *Morc2a*. (A) Gene structure of *Morc2a* and *Morc2b*. Coding regions are shown in black. Percent identities of nucleotide (nt) and amino acid (aa) sequences in the coding region are shown. 5’ and 3’ untranslated regions (UTRs) lack significant nt identity and are represented as open boxes. (B) Timing of the *Morc2b* retrotransposition. mya, million years ago. (C) Western blot analysis of MORC2A and MORC2B in lysates from adult mouse tissues. ACTB served as a loading control. (D, E) Expression and subcellular localization of MORC2B in adult testes. Immunofluorescence analysis of MORC2B was performed on frozen sections from wild type (D) and *Morc2b*^-/-^ (E) testes. DNA was stained with DAPI. Insets show enlarged view of boxed regions. Abbreviations: Pa, pachytene spermatocytes; Spc, spermatocytes; RS, round spermatids; ES, elongated spermatids; Sertoli, Sertoli cells. Scale bars, 25 μm. (F) RT-PCR expression analysis of *Morc2b* in juvenile testes. *Actb* serves as a control. (G) Quantification of *Morc2b* expression in juvenile testes by real-time PCR. The expression level of *Morc2b* was normalized to *Actb*. The relative expression level at postnatal day 20 was set at 1.0.

Sequence comparison of the five murine MORC members revealed that MORC2A and MORC2B exhibited the highest sequence homology within the family, with 73% amino acid sequence identity (S1A Fig). MORC2A and MORC2B were also the closest homologues according to phylogenetic analysis (S1B Fig). Both MORC2A and MORC2B contain the conserved GHKL-type ATPase and PHD zinc finger domains shared by MORC proteins (S2 Fig). The gene structure of *Morc2a* and *Morc2b* differs fundamentally: *Morc2a* contains 26 introns, whereas *Morc2b* lacks introns in the coding region (Fig 1A). This gene structure implies that *Morc2b* is a retrotransposed homologue of *Morc2a* that arose from reverse transcription of a processed transcript followed by integration into the genome. Although most retrotransposition events produce truncated or otherwise non-functional pseudogenes, a small number of retrotransposed genes have retained functionality [24, 25]. An annotated *Morc2a* gene (referred to as *Morc2* in non-rodent species) is found in more than 100 mammalian species in the NCBI database. In contrast, *Morc2b* is only present in mouse and rat but not in other non-rodent eutherians, suggesting that the *Morc2b* retrotransposition event occurred 12–24 million years ago prior to the radiation of mouse and rat (Fig 1B) [26–28]. Western blot analysis with a MORC2B-specific polyclonal antibody showed that MORC2B migrates at the predicted size of ~120 kDa (Fig 1C). MORC2B protein was abundant in testes but not detected in other adult mouse tissues examined, whereas MORC2A, migrating at the predicted size of ~120 kDa, was highly expressed in both testis and skeletal muscle (Fig 1C). Thus, the *Morc2b* gene encodes a bona fide testis-expressed protein and represents a functional retrotransposed gene rather than a pseudogene.

### Expression and localization of MORC2B in germ cells

We next examined the spatiotemporal localization pattern of MORC2B in adult testis. MORC2B was detected in germ cells with a distinct developmental-specific expression pattern but not in somatic cells such as Sertoli cells (Fig 1D and 1E). MORC2B was present in meiotic spermatocytes, abundant in post-meiotic haploid round spermatids, and absent from elongated spermatids (Fig 1D). MORC2B localized to the nucleus in spermatocytes and strongly to the nucleus in round spermatids. Absence of immunofluorescence signals in *Morc2b*-deficient spermatocytes supported the specificity of the MORC2B antibody (Fig 1E). The observed tissue- and cell type-specific expression of MORC2B and its stage-specific subcellular localization suggest a germ cell-specific nuclear function of *Morc2b*.

We further examined the expression of *Morc2b* using juvenile testes (day 8 through day 20) (Fig 1F and 1G). The first wave of spermatogenesis is synchronized [29]. At postnatal day 8, testes contain spermatogonia but no spermatocytes. Pre-leptotene and leptotene spermatocytes first appear at day 10, zygotene spermatocytes at day 12, pachytene spermatocytes at day 14, and round spermatids at day 20. *Morc2b* expression was absent prior to day 12, was detected at a low level at day 12, and increased significantly at day 14 and beyond (Fig 1F and 1G). This expression pattern was consistent with the immunofluorescence analysis of MORC2B in adult testis (Fig 1D). In conclusion, *Morc2b* is not expressed in spermatogonia, begins to express in zygotene spermatocytes at a low level, and increases expression from pachytene spermatocytes through round spermatids.

### *Morc2b* is essential for meiosis and fertility in both sexes

**Fig 2:**
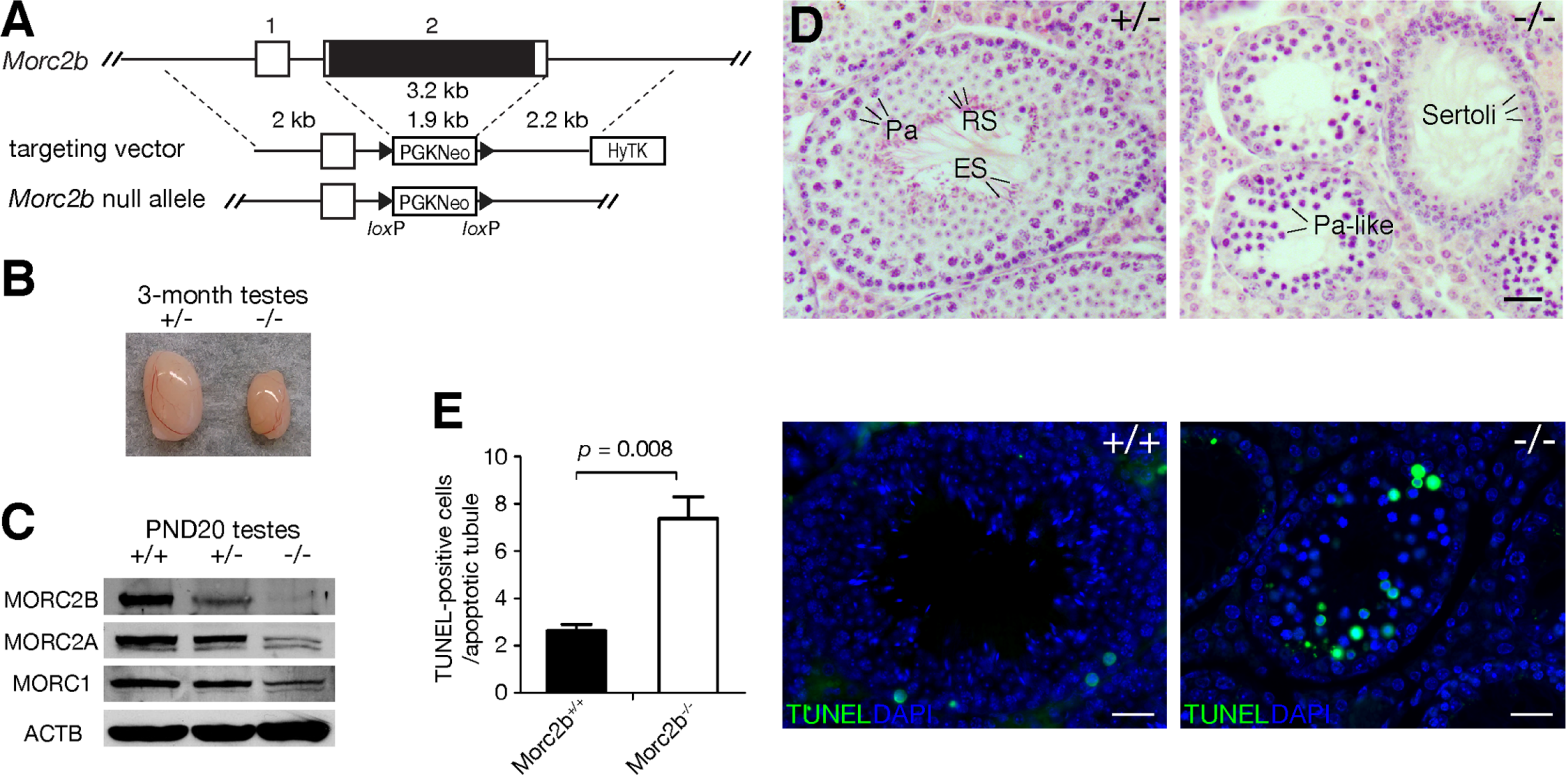
MORC2B is essential for male meiosis. (A) Targeted inactivation of the *Morc2b* gene. Deletion of exon 2 removes the entire coding region and thus results in a null allele. The neomycin selection marker PGKNeo is flanked by loxP sites. HyTK provides negative selection by ganciclovir. (B) Dramatic reduction in testis size in 3-month-old *Morc2b*^-/-^ males. (C) Western blot analysis of postnatal day 20 (PND20) *Morc2b*^+/-^ and *Morc2b*^-/-^ testes. ACTB serves as a loading control. (D) Histological analysis of 8-week-old *Morc2b*^+/-^ and *Morc2b*^-/-^ testes. Abbreviations: Pa, pachytene spermatocytes; RS, round spermatids; ES, elongated spermatids. Scale bars, 25 μm. (E) TUNEL analysis of seminiferous tubules from the testes of 8-week-old wild-type and *Morc2b*^-/-^ males. TUNEL-positive cells are shown in green. DNA was counterstained with DAPI. Scale bars, 25 μm. The graph on the left shows quantification of TUNEL-positive cells. Only tubule cross-sections with at least one TUNEL-positive cell (3 wild type testes and 3 *Morc2b*^-/-^ testes) were analyzed. 18 to 24 tubules per testis were counted. Statistical analysis was performed with Student’s *t*-test.

To assess the function of *Morc2b*, we generated a *Morc2b*-null allele by targeted deletion of exon 2 through homologous recombination in embryonic stem cells (Fig 2A). Exon 2 includes the entire *Morc2b* coding region. The offspring from intercrosses of heterozygous (*Morc2b*^+/-^) mice exhibited a normal Mendelian distribution of genotypes (wt, 72; *Morc2b*^+/-^, 119; *Morc2b*^-/-^, 65; χ^2^ test, *p* = 0.44), suggesting that *Morc2b* is dispensable for embryonic and postnatal development.

*Morc2b*^-/-^ mice were viable and appeared to be grossly normal. However, both *Morc2b*^-/-^ males and females were sterile. Adult males of all genotypes were of similar body weight (age 2–3 months; wt and *Morc2b*^+/-^, 26.0 ± 3.9 g; *Morc2b*^-/-^, 26.6 ± 3.6 g), but *Morc2b*^-/-^ males had significantly smaller testes than *Morc2b*^+/-^ control males (Fig 2B). *Morc2b*^-/-^ testes weighed approximately 70% less than control testes (*Morc2b*^-/-^, 53.4 ± 8.3 mg vs wt and *Morc2b*^+/-^, 166.4 ± 17.5 mg, n = 4, p = 0.0001). Western blot analysis confirmed reduced levels and absence of MORC2B protein in *Morc2b*^+/-^ and *Morc2b*^-/-^ testes respectively; MORC1 and MORC2A were present at reduced abundance in *Morc2b*^-/-^ testes (Fig 2C). Histological analysis of testes revealed that spermatogenesis in *Morc2b*^-/-^ males did not progress beyond meiotic stages. Seminiferous tubules of heterozygous (*Morc2b*^+/-^) testes contained germ cells at all stages including pachytene spermatocytes, round and elongating spermatids, whereas *Morc2b*-deficient tubules contained early meiotic germ cells including pachytene-like spermatocytes but were devoid of any post-meiotic spermatids (Fig 2D). TUNEL analysis revealed that apoptosis was strongly increased in *Morc2b*^-/-^ testes, suggesting that *Morc2b*-null spermatocytes were eliminated by apoptosis due to the activation of the pachytene checkpoint in response to meiotic defects (Fig 2E) [30, 31]. As expected, sperm were absent in *Morc2b*^-/-^ epididymides.

**Fig 3:**
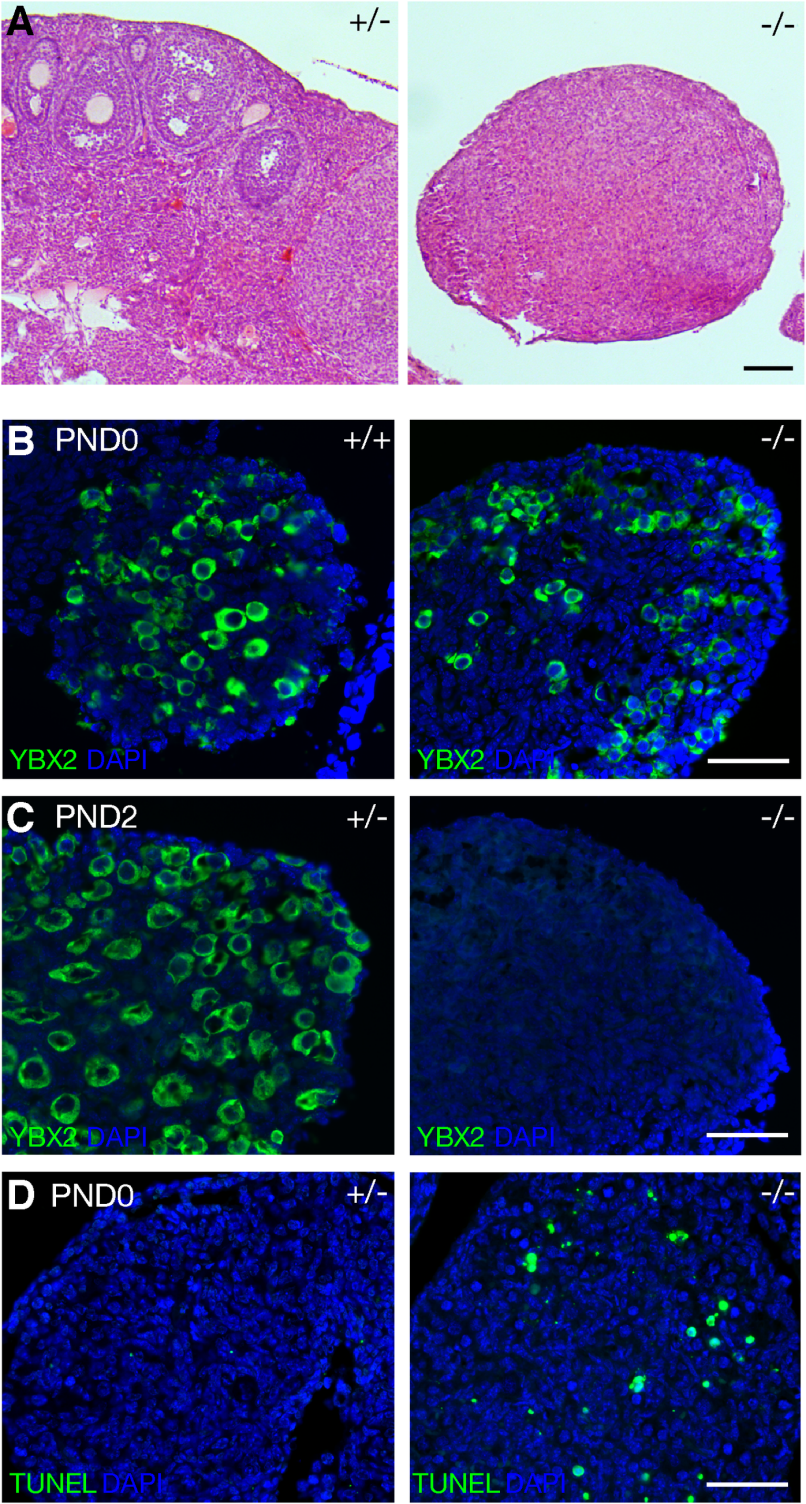
MORC2B is essential for oogenesis. (A) Histological analysis of the ovaries from 3-month-old *Morc2b*^+/-^ and *Morc2b*^-/-^ females. (B, C) Postnatal loss of oocytes in *Morc2b*^-/-^ ovaries. Frozen sections from postnatal day 0 (PND0) and 2 (PND2) ovaries were immunostained with anti-YBX2 antibodies. YBX2 is specifically expressed in oocytes [32]. (D) TUNEL analysis of PND0 ovaries. Scale bars: 100 μm (A), 50 μm (B-D).

The ovaries of adult *Morc2b*^-/-^ female mice were much smaller than those from heterozygous littermates and were devoid of oocytes (Fig 3A). To determine the time point of oocyte loss, we performed immunofluorescence analysis of ovaries with anti-YBX2 antibodies to label oocytes [32]. Oocytes were present in *Morc2b*^-/-^ ovaries at birth (Fig 3B) but disappeared by postnatal day 2 (Fig 3C). TUNEL analysis showed increased apoptosis of oocytes in *Morc2b*^-/-^ ovaries at birth (Fig 3D). Perinatal loss of oocytes was observed in several recombination-defective mouse mutants (*Dmc1*, *Msh5*, *Atm*, *Meiob*, or *Prdm9*) [16, 33, 34]. The early postnatal loss of oocytes in *Morc2b*^-/-^ mice therefore suggests severe defects in female meiosis. This data is consistent with the expression of *Morc2b* during meiosis in embryonic ovaries [16]. The *Morc2b* (previously referred to as *4932411A10Rik*) transcript is only present in embryonic ovaries at E13.5 and E14.5, but not at E15.5 and beyond including adulthood [16]. Collectively, our results show that *Morc2b* is essential for meiosis and fertility in both sexes.

### Failure of chromosomal synapsis in *Morc2b*^-/-^ germ cells

**Fig 4:**
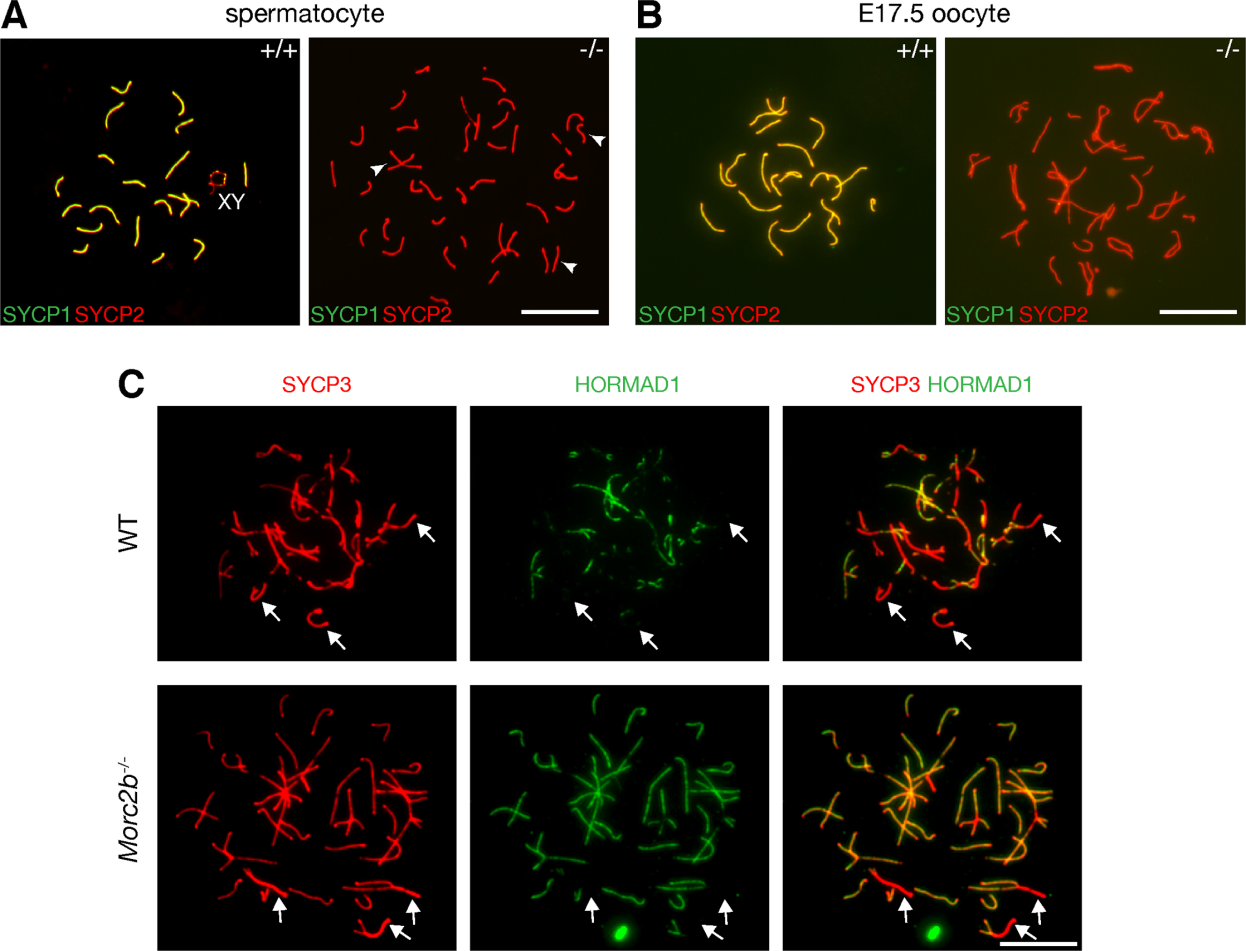
MORC2B is required for chromosomal synapsis in meiosis in both sexes. (A) Surface spread nuclei of spermatocytes from the testes of juvenile wild type and *Morc2b*^-/-^ males were immunostained for synaptonemal complex proteins (SYCP1 and SYCP2). The sex chromosomes in wild type pachytene spermatocyte (left) are labelled. Three paired chromosomes in *Morc2b*^-/-^ spermatocytes are indicated by arrowheads. (B) Surface spread nuclei of oocytes from wild type and *Morc2b*^-/-^ embryonic day 17.5 (E17.5) embryos were immunostained for SYCP1 and SYCP2. Note the apparent pairing and alignment of presumably homologous chromosomes judged by the equal length of SC axial elements in the *Morc2b-*deficient oocyte. (C) Surface spread nuclei of spermatocytes from wild type and *Morc2b*^-/-^ postnatal day 18 testes were immunostained for SYCP3 and HORMAD1. Arrows indicate synapsed regions. Scale bars, 10 μm.

**Fig 5:**
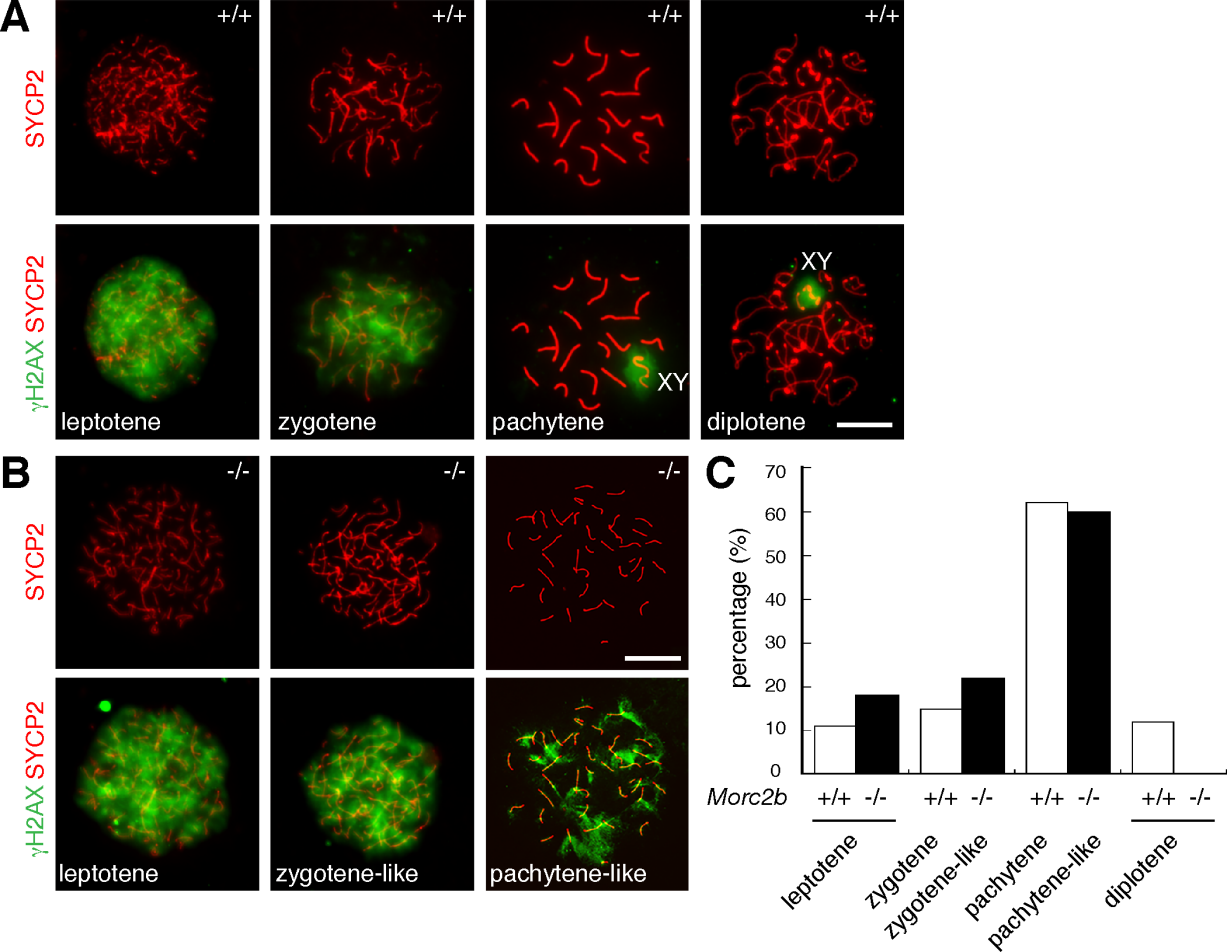
Persistence of γH2AX in *Morc2b*^-/-^ spermatocytes. (A, B) Spread nuclei of spermatocytes from wild type (A) and *Morc2b*^-/-^ (B) males at PND25 were immunostained with anti-SYCP2 and anti-γH2AX antibodies. Representative images of wild type spermatocytes at the leptotene through diplotene stages are shown. In *Morc2b*^-/-^ males, zygotene-like spermatocytes formed lateral elements and contained a high level of γH2AX, whereas pachytene-like spermatocytes showed more prominent lateral elements, alignment of lateral elements, and a low level of γH2AX. (C) Percentage of spermatocytes at meiotic stages (leptotene through diplotene). The number of spermatocytes analysed: wild type, 460; *Morc2b*^-/-^, 480. Scale bars, 10 μm.

We assessed chromosomal synapsis by immunofluorescence analysis of spread nuclei using antibodies against SYCP2, a component of synaptonemal complex (SC) axial elements, and SYCP1, a component of SC transverse elements [35, 36]. SC axial elements are formed at the leptotene stage, initiate synapsis through physical juxtaposition at the zygotene stage, achieve full synapsis on autosomes at the pachytene stage, and subsequently separate at the diplotene stage [37]. Wild-type pachytene spermatocytes contained fully synapsed autosomes, whereas the most advanced spermatocytes from *Morc2b*^-/-^ males were at a pachytene-like stage, characterized by apparent chromosome pairing and formation of SC axial elements (SYCP2) but absence of full chromosomal synapsis (Fig 4A). We quantified spermatocytes at different stages from juvenile wild type and *Morc2b*^-/-^ males and found that diplotene spermatocytes were absent in *Morc2b*^-/-^ males, indicating meiotic arrest at the pachytene-like stage (Fig 5).

We identified similar defects in meiotic progression in *Morc2b*^-/-^ oocytes (Fig 4B). Female germ cells enter meiosis shortly after sex determination during embryogenesis. At embryonic day 17.5 (E17.5), wild type pachytene oocytes had all 20 chromosome pairs fully synapsed, whereas *Morc2b*^-/-^ ovaries did not contain normal pachytene stage oocytes. The most advanced oocytes were at a pachytene-like stage as characterized by pairing and alignment of chromosomes and absence of extensive synapsis (Fig 4B). The defects in chromosomal synapsis were strikingly similar between *Morc2b*-deficient spermatocytes and oocytes. These results demonstrate that MORC2B is required for chromosomal synapsis during meiosis in both sexes.

HORMAD1 is associated with unsynapsed chromosomes [38–40]. In both wild type and *Morc2b*^-/-^ spermatocytes, HORMAD1 localized to the SC axial elements (SYCP3) of unsynapsed chromosomes but was excluded from synapsed regions (Fig 4C). This result is consistent with the synapsis defects in *Morc2b*^-/-^ mice.

### MORC2B is essential for meiotic recombination

To monitor meiotic recombination in *Morc2b*-deficient spermatocytes, we evaluated the formation of DNA double strand breaks (DSBs) and localization of recombination nodules in spread nuclei of *Morc2b*^-/-^ spermatocytes. During meiosis, following PRDM9-mediated chromatin changes at recombination hotspots, the SPO11 protein catalyses the formation of DSBs, which elicits the DNA damage response [19, 21, 41–43], leading to activation of the ATM kinase and subsequent phosphorylation of H2AX (termed γH2AX). At the leptotene and zygotene stages, γH2AX is present on autosomal chromatin and distributed widely throughout the nucleus (Fig 5A). The presence of strong γH2AX signals suggested that DSBs are formed in *Morc2b*^-/-^ leptotene and zygotene spermatocytes (Fig 5B). In normal spermatocytes, γH2AX disappears from the autosomes following meiotic DSB repair and becomes restricted to the XY chromatin during the pachytene and diplotene stages, concomitant with meiotic sex chromatin inactivation (Fig 5A). However, the pachytene-like *Morc2b*^-/-^ spermatocytes remained γH2AX-positive throughout the nucleus and failed to form a sex body (Fig 5B), suggesting a failure in the repair of meiotic DSBs.

**Fig 6:**
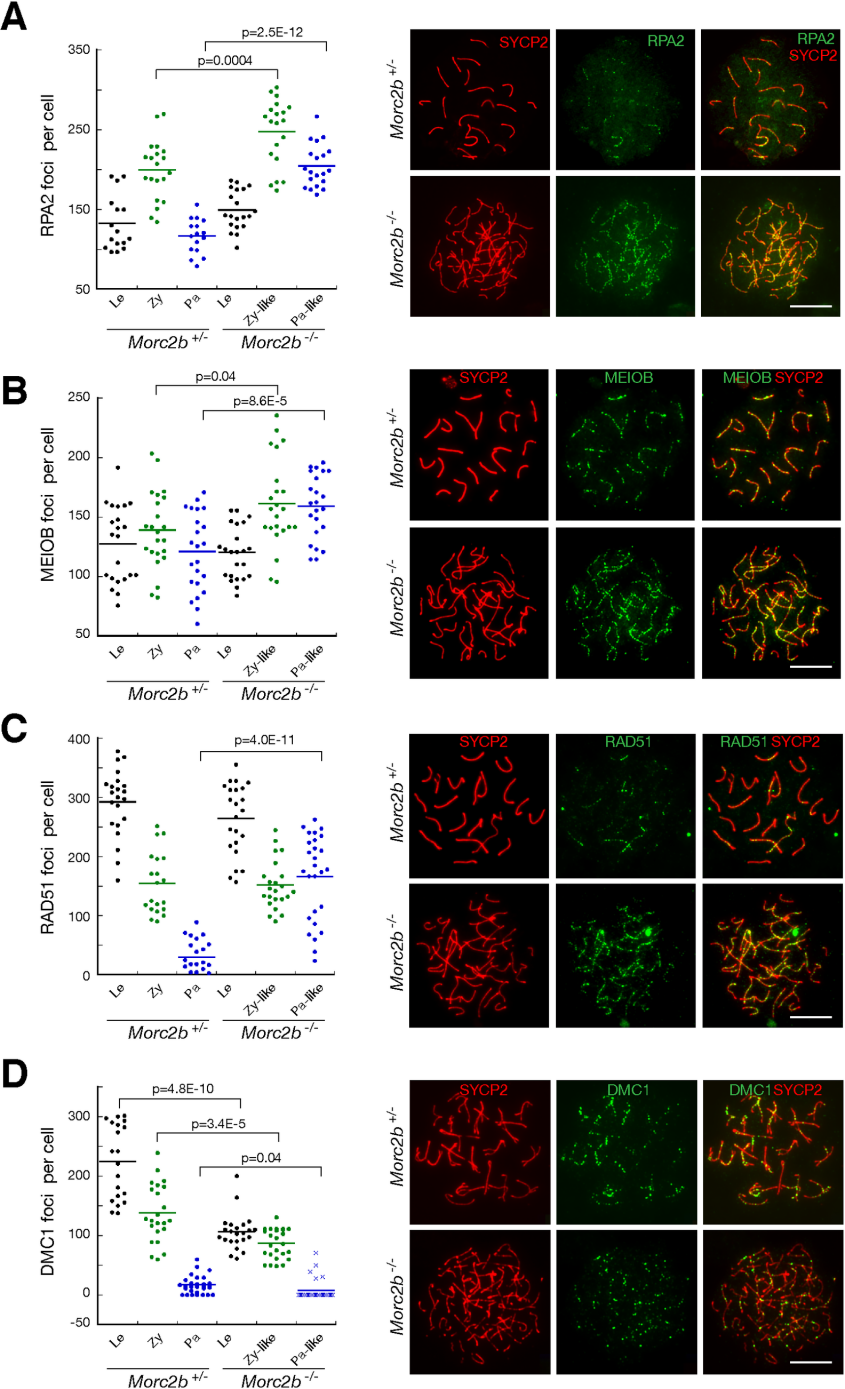
MORC2B is essential for meiotic recombination. Immunofluorescence was performed on spread nuclei of spermatocytes from *Morc2b*^+/-^ and *Morc2b*^-/-^ testes at postnatal day 18. Based on the morphology of the synaptonemal complex (SYCP2 immunolabelling), spermatocytes were categorized into the following stages: leptotene (Le), zygotene (Zy), pachytene (Pa), zygotene-like (Zy-like), and pachytene-like (Pa-like). Each dot represents the number of DNA repair protein foci per cell. Solid lines show the average number of foci for each category of spermatocytes. (A) RPA2 foci. (B) MEIOB foci. (C) RAD51 foci. (D) DMC1 foci. Representative images of spermatocytes at pachytene or pachytene-like stages are shown (A-C). Spermatocytes at zygotene and zygotene-like stages are shown in D. Scale bars, 10 μm. Statistics was performed by Student’s *t* test.

Meiotic recombination is executed through coordinated actions of a large number of DNA repair proteins [37]. We examined four single-stranded DNA-binding proteins: RPA, MEIOB, RAD51, and DMC1 (Fig 6). These recombination proteins form distinct foci on meiotic chromosomes. The RPA heterotrimer consists of RPA1, RPA2, and RPA3. RPA binds to ssDNA ends of the meiotic DSBs [44]. MEIOB forms a heterodimer with SPATA22 and interacts with RPA [34, 45, 46]. RAD51 and DMC1 are recombinases. RAD51 and DMC1 form filaments on RPA-coated ssDNA and direct strand invasion into the homologous chromosome, which is required for crossover formation, homologue pairing, and chromosomal synapsis [47]. Consistent with the formation of DSBs, unsynapsed chromosomes in *Morc2b*^-/-^ spermatocytes contained abundant foci of these recombination proteins (Fig 6). The initial number of RPA2, MEIOB, and RAD51 was similar between *Morc2b*^+/-^ and *Morc2b*^-/-^ spermatocytes at the leptotene stage. With the progression of meiotic recombination, the number of RPA2 and RAD51 foci decreased progressively in control (*Morc2b*^+/-^) spermatocytes, however, the number of RPA2 foci was sharply higher in *Morc2b*^-/-^ spermatocytes at both zygotene-like and pachytene-like stages (Fig 6A) and the number of RAD51 foci was higher at the pachytene-like stage (Fig 6C). The number of MEIOB foci also increased in *Morc2b*^-/-^ spermatocytes at the zygotene-like and pachytene-like stages (Fig 6B). These defects further suggested that meiotic DSBs were not repaired in the absence of MORC2B. In contrast, the number of DMC1 foci decreased significantly in *Morc2b*^-/-^ spermatocytes, suggesting defects in strand invasion and/or stabilization of homologue pairing (Fig 6D). Such defects were consistent with the failure in chromosomal synapsis in *Morc2b*-deficient germ cells (Fig 4). Furthermore, we did not detect MLH1 foci, representing sites of future crossovers, in *Morc2b*^-/-^ spermatocytes. These results demonstrate that MORC2B is required for meiotic recombination.

### Lack of change in the levels of retrotransposons and H3K4me3 in *Morc2b*^-/-^ testes

Loss of MORC1 causes upregulation of retrotransposons in male germ cells [13]. We examined the expression of LINE1 and IAP retrotransposons in *Morc2b*^-/-^ testes. In contrast with the upregulation of LINE1 and IAP in *Mov10l1*^-/-^ testes (positive control) [48], retrotransposons were not de-silenced in *Morc2b*^-/-^ testes (S3 Fig), implying functional divergence of these two MORC family members.

PRDM9 catalyzes trimethylation of H3K4 and consequently loss of PRMD9 reduces the level of H3K4me3 in male meiotic germ cells [16]. We confirmed the reduced level of H3K4me3 in spermatocytes from *Prdm9*^-/-^ testes (S4 Fig). *Morc2b* was reported to be a PRDM9 target gene [16]. We found that the H3K4me3 level was comparable in spermatocytes between wild type and *Morc2b*^-/-^ males (S4 Fig), suggesting that loss of MORC2B is not responsible for reduced H3K4me3 in *Prdm9*^-/-^ testes.

### Misregulated expression of meiosis-specific genes in *Morc2b*^-/-^ testes

**Fig 7:**
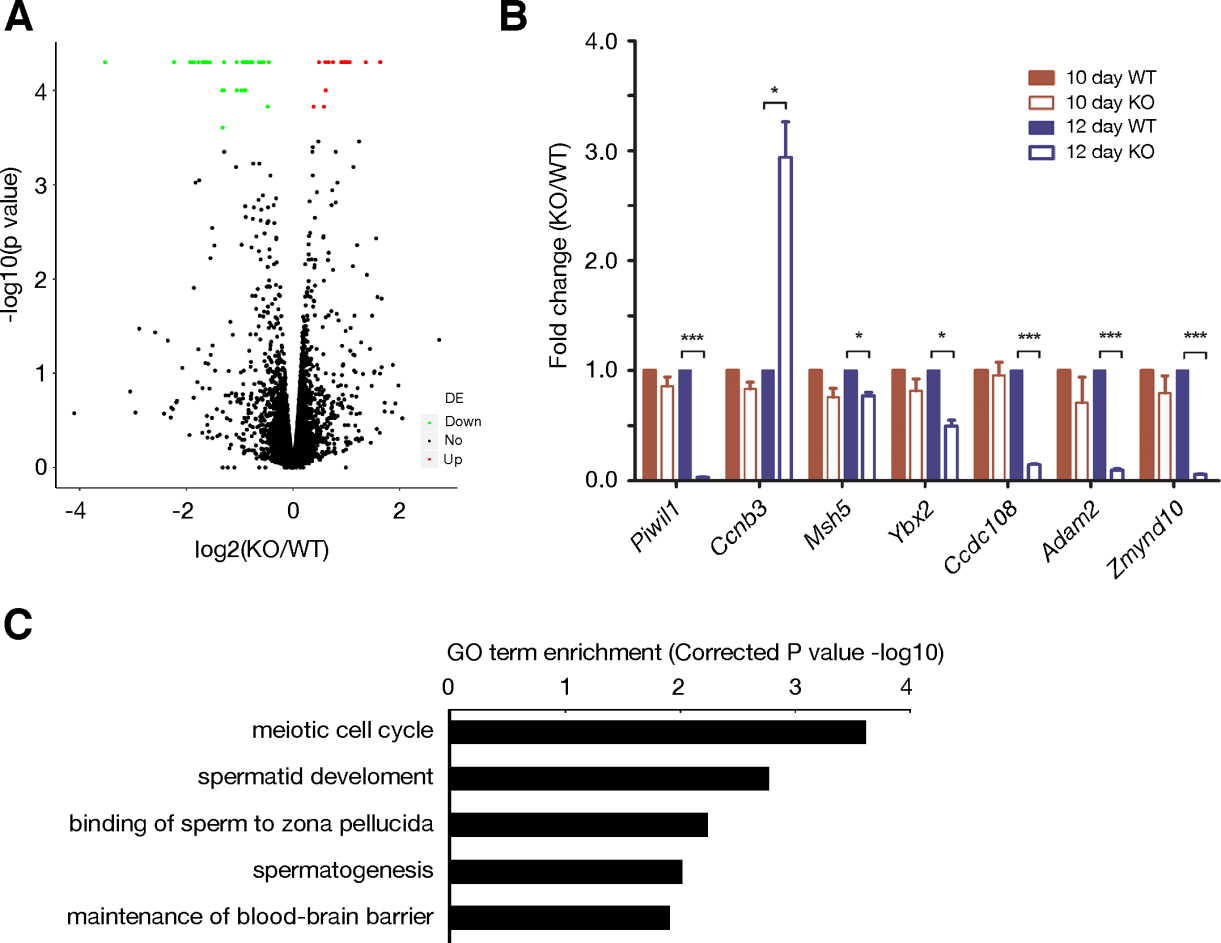
Transcript profiling analysis of wild type and *Morc2b*^-/-^ testes. (A) Volcano plot of transcript levels between wild type and *Morc2b*^-/-^ testes at postnatal day 12. The expression cut-off is at least 1 FPKM in either wild type or *Morc2b*^-/-^ testes. The differentially expressed genes (FDR < 0.05) are highlighted in red (upregulated in *Morc2b*^-/-^) and green (downregulated in *Morc2b*^-/-^). (B) Validation of differentially expressed genes by real-time PCR analysis. Statistics was performed with Student’s *t*-test: *, p<0.05; **, p<0.01; ***, p<0.001. (C) GO term enrichment in differentially expressed genes.

Since the MORC family proteins are involved in chromatin remodelling, we sought to examine the transcriptome in *Morc2b*^-/-^ testes by RNA-seq. We chose testes at postnatal day 12 for two reasons. First, *Morc2b* begins its expression at day 12 (Fig 1G). Second, the histology of testes is comparable between wild type and *Morc2b*^-/-^ males at day 12, when the most advanced germ cells are at the zygotene stage. Analysis of RNA-seq data identified 71 differentially expressed genes: 57 downregulated and 14 upregulated in *Morc2b*^-/-^ testes (Fig 7A and S1 Table). Seven genes (six downregulated and one upregulated) were chosen for validation by real-time PCR. The differential expression was confirmed for all seven genes at day 12 (Fig 7B). As expected, their expression was comparable between wild type and *Morc2b*^-/-^ testes at day 10 (Fig 7B). Gene ontology analysis identified meiotic cell cycle as the most affected biological process (Fig 7C). Interestingly, two meiosis-specific genes *Msh5* and *Ccnb3* are downregulated and upregulated respectively. MSH5, a DNA repair protein, is required for chromosomal synapsis [49, 50]. CCNB3 is a meiosis-specific cyclin [51]. These data strongly suggest that mis-regulated expression of meiosis-specific genes may contribute to the meiotic defects in *Morc2b*^-/-^ mice.

### MORC2B interacts with MORC2A

**Fig 8:**
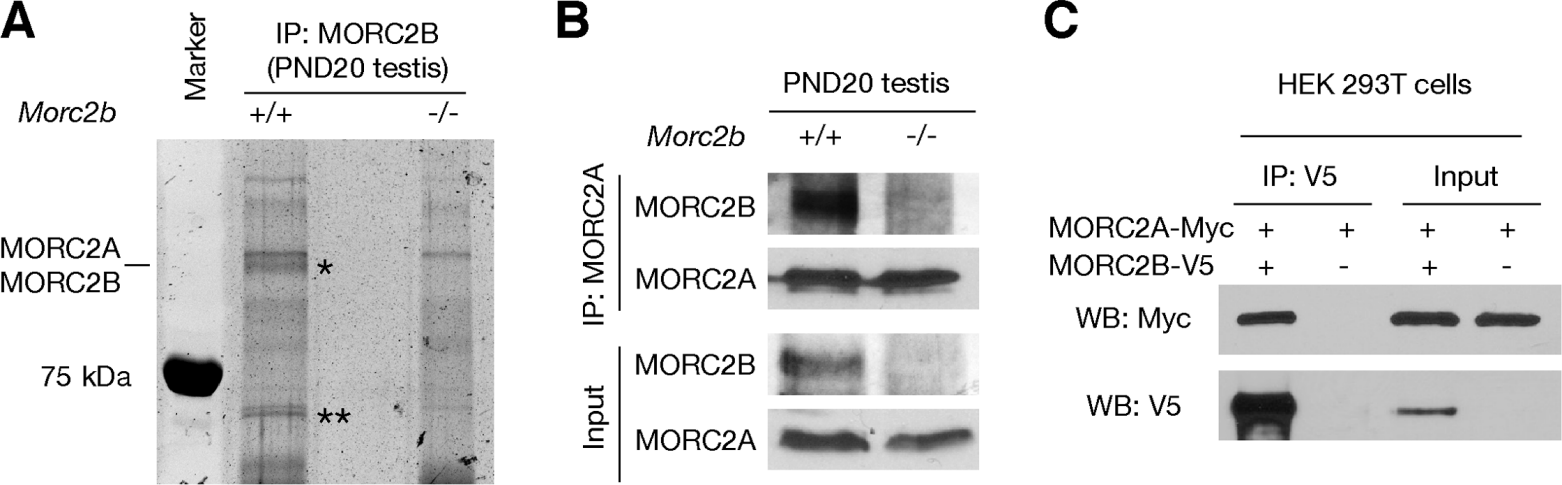
MORC2B interacts with its paralogue MORC2A. (A) Identification of MORC2B-associated proteins in lysates from PND20 mouse testes by IP and mass spectrometry. The gel was stained with SYPRO Ruby. Gel slices corresponding to the two unique protein bands present in wild-type but not in *Morc2b*^-/-^ testes (*~110 kDa; ** ~65 kDa) were cut from both lanes and subject to mass spectrometry. Proteins with at least three unique peptides are provided in S2 and S3 Tables. (B) Co-IP analysis of MORC2A and MORC2B from PND20 testicular protein extracts. (C) Co-IP analysis of MORC2A and MORC2B in HEK 293T cells. Expression constructs for tagged MORC2A and MORC2B were transfected into HEK 293T cells alone or together as indicated, followed by co-IP with anti-V5 antibody and western blotting with antibodies as indicated. Input was 10% of the lysate used for IP.

To identify MORC2B-interacting partners, we performed immunoprecipitation (IP) with testicular protein extracts using the MORC2B antibody. Two protein bands were present in immunoprecipitated proteins from wild type testis but not detected in *Morc2b*^-/-^ testis IP (Fig 8A). The upper band had the same apparent molecular weight as MORC2B and contained both MORC2A and MORC2B and three other proteins (Fig 8A and S2 Table). Mass spectrometry of the lower unique band identified three proteins: DDX41, HSP72, and ARID3B (S3 Table). We verified the association of MORC2A and MORC2B in testes by co-IP and western blot analyses and confirmed that MORC2B was present in the immunoprecipitated proteins from wild type testes with anti-MORC2A antibody (Fig 8B). Additionally, co-expression and co-IP in HEK 293T cells also validated the association between MORC2A and MORC2B (Fig 8C). Our results suggest that MORC2B may regulate meiosis through interaction with MORC2A.

## Discussion

The MORC family proteins are involved in chromatin remodelling, transcriptional regulation, and transposon silencing. Here we find that *Morc2b* is required for meiosis and fertility in both males and females. Interestingly, the *Morc2b* gene evolved in the rodents via retrotransposition from *Morc2a*. Strikingly, the relatively young *Morc2b* gene has evolved an essential role in meiosis and fertility, suggesting a strong selection pressure.

Among the *Morc* gene family, *Morc1* and *Morc2b* are germ cell-specific but exhibit distinct functions. This is evident from differences in the phenotype of the corresponding mouse mutants. The fertility of *Morc1* mutant is sexually dimorphic: males are sterile but females are fertile [11, 12], whereas inactivation of *Morc2b* causes sterility in both sexes. The *Morc1* mutant phenotype is similar to that of piRNA (Piwi-interacting RNA) pathway mutants: male-only sterility and de-repression of transposable elements (LINE1 and IAP) in male germ cells [13, 52]. Since the piRNA pathway appears to be intact in *Morc1* mutant germ cells, MORC1 most likely protects genome integrity in male germ cells by silencing transposable elements through a different yet unknown mechanism [13]. In contrast, MORC2B deficiency does not cause de-silencing of LINE1 and IAP retrotransposons in testes (S3 Fig) but leads to a failure in chromosomal synapsis and meiotic recombination in both sexes.

Human MORC2 plays a critical role in chromatin remodelling in DNA damage response and transcriptional gene silencing [3–6]. MORC2 acts as a transcriptional repressor of the *CAIX* gene (carbonic anhydrase IX) by decreasing histone H3 acetylation at the *CAIX* promoter. MORC2 binds to the *CAIX* promoter and recruits HDAC4 (histone deacetylase 4) to deacetylate histone H3, which is associated with a repressed chromatin state [3]. Similarly, MORC2 represses p21 in gastric cancer cells by recruiting HDAC1 to the p21 promoter [4]. MORC2 also modulates chromatin configuration during the DNA damage response [5, 6]. Upon DNA damage, MORC2 becomes phosphorylated by p21-activated kinase 1 (PAK1), exhibits DNA-dependent ATPase activity, and facilitates chromatin relaxation [5]. Given the known function of MORC proteins in chromatin remodelling, MORC2B might play a role in the regulation of high-order chromatin structure during meiosis.

Loss of MORC2B results in mis-expression of 71 genes in testes. Out of the 71 genes, 30 genes have been disrupted in mice (S1 Table). Twelve knockout mice exhibit sterility or impaired fertility: *Adam2*, *Adam3*, *Clgn*, *Crisp1*, *Fmr1*, *Krt8*, *Msh5*, *Piwil1*, *Rsph1*, *Tnp1*, *Tnp2*, and *Ybx2*. The remaining 18 knockout mice exhibit lethality, or no defects, or somatic defects but normal fertility. Several affected genes are known to play critical roles in meiosis: *Msh5*, *Fmr1*, and *Ccnb3*. *Msh5* is downregulated in *Morc2b*^-/-^ testes, whereas *Fmr1* and *Ccnb3* are upregulated in *Morc2b*^-/-^ testes. MSH5 forms a heterodimer with MSH4 and functions in meiotic recombination. Inactivation of *Msh5* causes a failure in chromosomal synapsis and thus meiotic arrest [49, 50]. FMR1 localizes to chromatin and regulates DNA damage response [53]. CCNB3 (cyclin B3) is specifically expressed in leptotene and zygotene spermatocytes [51]. Strikingly, mis-expression of the human *CCNB3* transgene in mouse spermatocytes after the zygotene stage disrupts spermatogenesis [54]. Therefore, mis-expression of these meiosis genes could contribute to the meiotic defects in *Morc2b*^-/-^ mice. It is possible that MORC2B regulates the transcription of these genes through chromatin relaxation or epigenetic modifications.

Our biochemical studies demonstrate that MORC2B interacts with MORC2A. The interaction among MORC proteins is also present in *Arabidopsis*. AtMORC6 interacts with AtMORC1 and AtMORC2 in two mutually exclusive protein complexes [55]. Both AtMORC1 and AtMORC2 are needed to repress the set of genomic loci silenced by AtMORC6. The interaction between MORC proteins and the non-redundant nature of their functions are conserved between *Arabidopsis* and mouse and possibly so in other species. In addition, MORC2B may function through other interacting proteins such as ARID3B –a member of the ARID (AT-rich interaction domain) family of DNA-binding proteins (S3 Table) [56, 57]. Genetic studies of MORC2A and ARID3B in germ cells are not available yet but are necessary to determine their functional requirement for meiosis and the physiological significance of their interaction with MORC2B. As DNA-dependent ATPases, MORC proteins have been found to modulate chromatin superstructure in DNA damage response, heterochromatin formation, and gene silencing. Further studies are necessary to elucidate a possible role of MORC2B in chromatin remodelling in the regulation of meiosis.

PRDM9, a meiosis-specific histone H3 methyltransferase, is a major determinant of meiotic recombination hotspots in mice, primates, and humans [19–21]. PRDM9 binds to recombination hotspots in a sequence-specific manner through its variable number of zinc fingers. Disruption of *Prdm9* results in meiotic failure and sterility in both males and females [16]. The cause of meiotic failure in *Prdm9*-null mice has not been identified, and the relationship between control of meiotic recombination hotspots and meiotic progression remains unclear. While PRDM9 catalyses H3K4me3 at hotspots, it also affects the expression of one target gene–*Morc2b*. In *Prdm9*-deficient testes, *Morc2b* is not expressed [16]. *Morc2b* expression is also nearly absent in sterile hybrids of mouse subspecies [17]. Furthermore, both *Morc2b* and *Prdm9* mutants exhibit a failure in chromosomal synapsis and meiotic recombination. The similar phenotype of these two mutants raises the intriguing possibility that the absence of *Morc2b* might be responsible for or at least contribute to the meiotic failure in *Prdm9*-null mice.

## Materials and methods

### Ethics statement

Mice were maintained and used for experimentation according to the protocol approved by the Institutional Care and Use Committee of the University of Pennsylvania.

### Antibody production

The mouse MORC2B C-terminal fragment (aa 823–1022) and MORC1 C-terminal fragment (aa 751–950) were expressed as GST fusion proteins in *E*. *coli* using the pGEX4T-1 vector and affinity purified with glutathione sepharose. Two rabbits were immunized with each fusion protein (Cocalico Biologicals Inc.). The resulting working antisera are: anti-MORC2B, UP2419 and UP2420; anti-MORC1, UP2424. Affinity-purified antibodies were used for immunofluorescence analysis and Western blotting. The following additional antibodies were used: MORC2A (1:250, catalogue number PAB15729, Abnova) and ACTB (1:7,500, catalogue number A5441, clone AC-15, Sigma). The MORC2A antibody (Abnova) was produced against its C-terminal fragment (aa 791–1030), which displays 69% aa sequence identity with MORC2B. The MORC1 antigen (aa 751–950) shows 30% aa sequence identity with MORC2B.

### Targeted inactivation of the *Morc2b* gene

In the targeting vector, the 3.2-kb *Morc2b* coding exon was replaced with the PGKNeo selection cassette (Fig 2A). The two homologous arms were amplified from a *Morc2b*-containing BAC clone (RP24-63E7) by high-fidelity PCR. The HyTK negative selection cassette was cloned after the right arm. V6.5 embryonic stem (ES) cells (on a C57BL/6 x 129S4/SvJae hybrid background) were electroporated with the ClaI-linearized targeting vector. ES cells were cultured in the presence of 350 μg/ml G418 and 2 μM ganciclovir. Out of 96 double-resistant ES cell clones, nine targeted clones were identified by long-distance PCR. Clone 1F3 was injected into blastocysts. Germline transmission of the knockout allele was obtained through breeding of chimera males with C57BL/6 females. Offspring of intercrosses of *Morc2b*^+/-^ mice were used for all the analyses. Wild-type allele (220 bp) was assayed by PCR with primers TGCACTGAACCCGACACTAC and GGTAGGAGCGGCAGAGATTC. The *Morc2b* mutant allele (415 bp) was assayed by PCR with primers ATAGCAGGCATGCTGGGGATGCGGT and TGCACCTACACCAGGCAGCTCAGG.

### Histological, immunofluorescence, and surface nuclear spread analyses

For histological analysis, testes and ovaries were fixed in Bouin’s solution, embedded with paraffin, and sectioned. Sections were stained with haematoxylin and eosin. Color histological images were captured on a Leica DM5500B microscope with a DFC450 digital color camera (Leica Microsystems). For immunofluorescence and TUNEL analysis, testes and ovaries were fixed in 4% paraformaldehyde for 3 h or overnight at 4°C, dehydrated, embedded, and sectioned using a cryostat. TUNEL assays were performed with the ApopTag Fluorescein In Situ Apoptosis Detection Kit (Catalogue number S7110, EMD Millipore). Nuclear spread analysis of spermatocytes and oocytes was performed as previously described [58, 59]. The following antibodies were used for immunofluorescence: SYCP1 (1:50, catalogue number ab15090, Abcam), SYCP2 [35], SYCP3 (1:200, catalogue number ab97672, Abcam), HORMAD1 [39], γH2AX (1:500, catalogue number 16-202A, Clone JBW301, EMD Millipore), RPA2 (1:100, catalogue number 2208S, clone 4E4, Cell Signaling Technology), MEIOB [34], RAD51 (1:30, catalogue number sc-8349 H-92, Santa Cruz Biotechnology), DMC1 (1:30, catalogue number sc-22768 H-100, Santa Cruz Biotechnology). FITC- and Texas red-conjugated secondary antibodies were used. Fluorescence images were captured with an ORCA Flash4.0 digital monochrome camera (Hamamatsu Photonics) on a Leica DM5500B microscope (Leica Microsystems).

Sections of postnatal day 14 testes (wild type, *Morc2b*^-/-^, and *Prdm9*^-/-^) were immunostained with H3K4me3 antibody (1:200, catalogue number ab8580, Abcam). *Prdm9* targeted mice were obtained from Jackson Laboratory (Stock No: 010719) [16]. Images were acquired under the same condition. The relative intensity of H3K4me3 fluorescence signal was quantified using the ImageJ software. One pachytene or pachytene-like spermatocyte and one Sertoli cell were randomly selected from each tubule cross-section (10 tubules/genotype). The relative H3K4me3 signal intensity in the spermatocyte was normalized to that in the Sertoli cell.

### RNA-seq, DESeq analysis, and real-time PCR validation

Total RNA was isolated from eight pairs of postnatal day 12 mouse testes (~16 mg/pair; 4 pairs of wild type and 4 pairs of *Morc2b*^-/-^) using TRIzol reagents (Thermo Fisher Scientific). The RNA concentration was determined using a NanoDrop 2000 Spectrophotometer (Thermo Fisher Scientific). Equal amounts (1 μg) of total RNA from each sample were used to generate RNA-seq libraries using TruSeq Stranded mRNA Library Preparation Kit Set A (Cat. No. RS-122-2101, Illumina) according to the manufacturer’s instruction. The concentration of DNA library templates was determined using a Qubit 3.0 Fluorometer (Thermo Fisher Scientific). The quality of libraries was evaluated using the Agilent 4200 TapeStation (Agilent Technologies). Eight individual libraries (4 wild type and 4 *Morc2b*^-/-^) were pooled in equal amounts for sequencing using the Illumina NextSeq 500/550 High Output v2 kit (Illumina, 75 cycles, FC-404-2005) and the NextSeq 500 system (Illumina). The RNA-seq data are available under GEO accession no: GSE103127.

After trimming the adapter sequences and removing the low-quality reads, the clean reads were mapped to the mouse reference genome (NCBI37/mm9) using Tophat with default parameters. Mapped reads were subjected to Cufflinks to estimate gene expression levels [60]. The expression of each gene was normalized by calculating fragments per kilobase of exon per million fragments mapped (FPKM). The FPKM values of each gene for both wild type and *Morc2b*^-/-^ group were used to assess the differential expression with Cuffdiff. The expression cutoff of ≥ 1FPKM in either wild type or *Morc2b*^-/-^ testes was applied. Differentially expressed genes were determined by an adjusted P value (false discovery rate, FDR) < 0.05 based on Benjamini and Hochberg multiple testing correction. A volcano plot was constructed to illustrate the differentially expressed genes by plotting log2 of the fold change on the X axis and the negative log10 of the p value on the Y axis (Fig 7A).

The expression of seven differentially expressed genes was analyzed using independent testis samples from postnatal day 10 and 12 mice (3 testis samples per genotype per time point) by real-time PCR. Expression of LINE1 and IAP retrotransposons in wild type and *Morc2b*^-/-^ testes at postnatal day 14 was assayed by real-time PCR (S3 Fig). Postnatal day 14 *Mov10l1*^-/-^ testes were used as a positive control for de-silencing of LINE1 and IAP [48, 61]. Real-time PCR primers are listed in S4 Table. Each sample was assayed in triplicates. Quantification was normalized to *Actb* using the Ct method (ABI Prism 7700 Sequence Detection System, Applied Biosystems).

### Co-immunoprecipitation, mass spectrometry, and transfection constructs

Co-immunoprecipitation was performed with postnatal day 20 mouse testes using affinity-purified MORC2B antibodies as previously described [34]. Immunoprecipitated proteins were resolved by SDS-PAGE. The protein bands specific to the wild type testis sample were subjected to mass spectrometry for protein identification.

The full-length coding sequences of mouse *Morc2a* and *Morc2b* were cloned into pcDNA3.1/myc-His vector and pcDNA3.1/V5-His-TOPO vector respectively. Plasmids were transfected into HEK 293T cells. Forty-eight hours after transfection, cells were collected and lysed in whole cell lysis buffer (50 mM HEPES, pH 7.5, 140 mM NaCl,1 mM DTT, 10% glycerol, 0.5% NP-40, 1 mM PMSF). Immunoprecipitation on protein lysate was performed with anti-V5 antibody (Catalogue number R96025, Invitrogen), followed by Western blotting with anti-Myc antibody (Catalogue number 631206, Clontech).

### Statistics

Statistical analysis was performed with Student’s *t*-test or χ^2^ test.

## Supporting information

S1 Fig Phylogenetic analysis of mouse MORC proteins. (A) Pairwise amino acid sequence identity between mouse MORC proteins. (B) Phylogenetic tree of MORC proteins. A Neighbour Joining tree was built using the BLOSUM62 matrix. MORC protein accession numbers were as follows: MORC1, NP_034946; MORC2A, NP_001152760; MORC2B, NP_808387; MORC3, NP_001038994; MORC4, NP_001180238.
(TIF)

S2 Fig Alignment of mouse MORC proteins. Multiple sequence alignment was performed using CLUSTAL Omega (v1.2.1). Protein sequence accession numbers were as shown in S1 Fig. The GHKL-type ATPase and PHD zinc finger domains are highlighted. Identical and similar residues are indicated by asterisks and semicolons, respectively.
(DOCX)

S3 Fig Quantitative RT-PCR expression analysis of LINE1 and IAP in wild type and *Morc2b*^-/-^ testes at postnatal day 14. Postnatal day 14 *Mov10l1*^-/-^ testes were used as positive controls for LINE1 and IAP de-silencing. *, statistically significant; ns, non-significant.
(TIF)

S4 Fig Analysis of H3K4me3 level in wild type, *Morc2b*^-/-^, and *Prdm9*^-/-^ testes at postnatal day 14. (A) Testis sections were immunostained with anti-H3K4me3 antibody. DNA was stained with DAPI. Scale bars, 25 μm. (B) Quantification of H3K4me3 fluorescence. The fluorescence in the most advanced spermatocytes (wild type, pachytene; mutant, pachytene-like) and Sertoli cells were quantified using Image J. The Y axis shows the relative intensity (spermatocyte/Sertoli cell). *, statistically significant (p < 0.05, Student’s *t*-test); ns, non-significant.
(TIF)

S1 Table List of differentially expressed genes between wild type and *Morc2b*^-/-^ testes at postnatal day 12. (XLSX)

S2 Table Unique proteins identified in the ~110 kDa band present in wild type but not *Morc2b*^-/-^ testis IP. (XLSX)

S3 Table Unique proteins identified in the ~65 kDa band present in wild type but not *Morc2b*^-/-^ testis IP. (XLSX)

S4 Table Real-time PCR primers. (XLSX)
