# Supplementary figures and images for: MORC2B is essential for meiotic progression and fertility
Source: PLoS Genet. 2018 Jan 12;14(1):e1007175. doi: 10.1371/journal.pgen.1007175 (PMC5785033; doi:10.1371/journal.pgen.1007175)

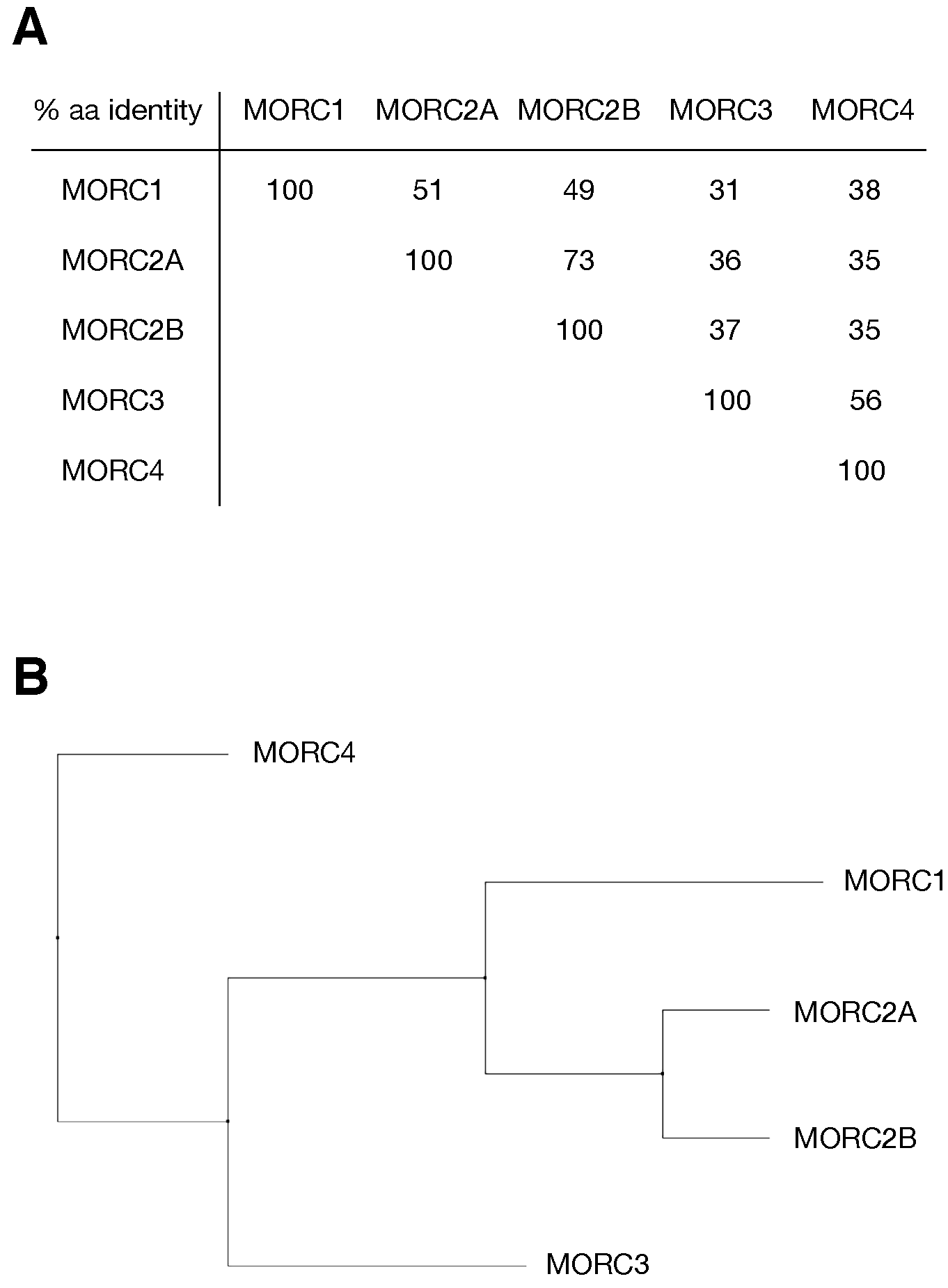

Supplement: S1 Fig — (A) Pairwise amino acid sequence identity between mouse MORC proteins. (B) Phylogenetic tree of MORC proteins. A Neighbour Joining tree was built using the BLOSUM62 matrix. MORC protein accession numbers were as follows: MORC1, NP_034946; MORC2A, NP_001152760; MORC2B, NP_808387; MORC3, NP_001038994; MORC4, NP_001180238. (TIF) [file pgen.1007175.s001.tif]

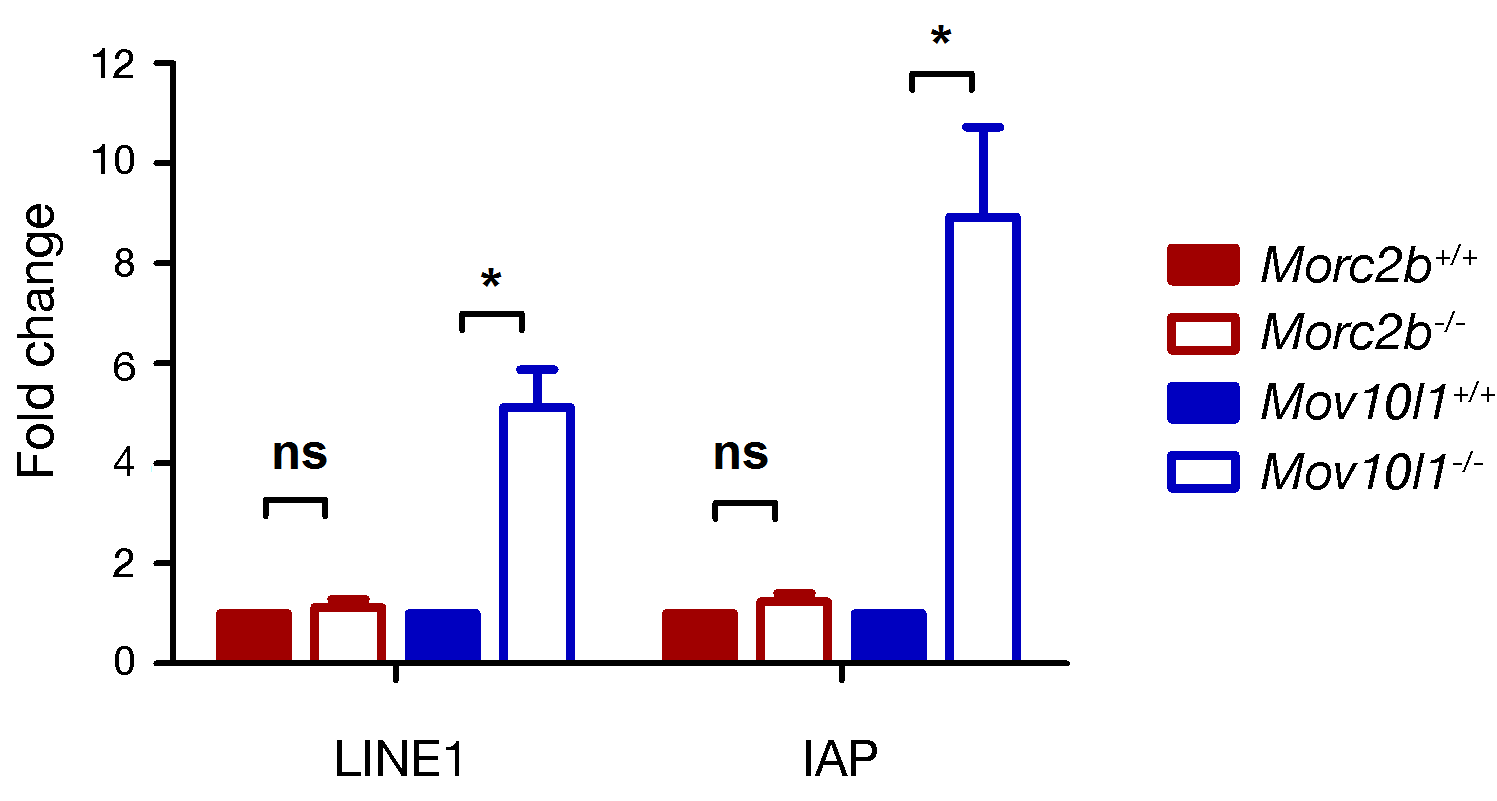

Supplement: S3 Fig — Postnatal day 14 Mov10l1-/- testes were used as positive controls for LINE1 and IAP de-silencing. *, statistically significant; ns, non-significant. (TIF) [file pgen.1007175.s003.tif]

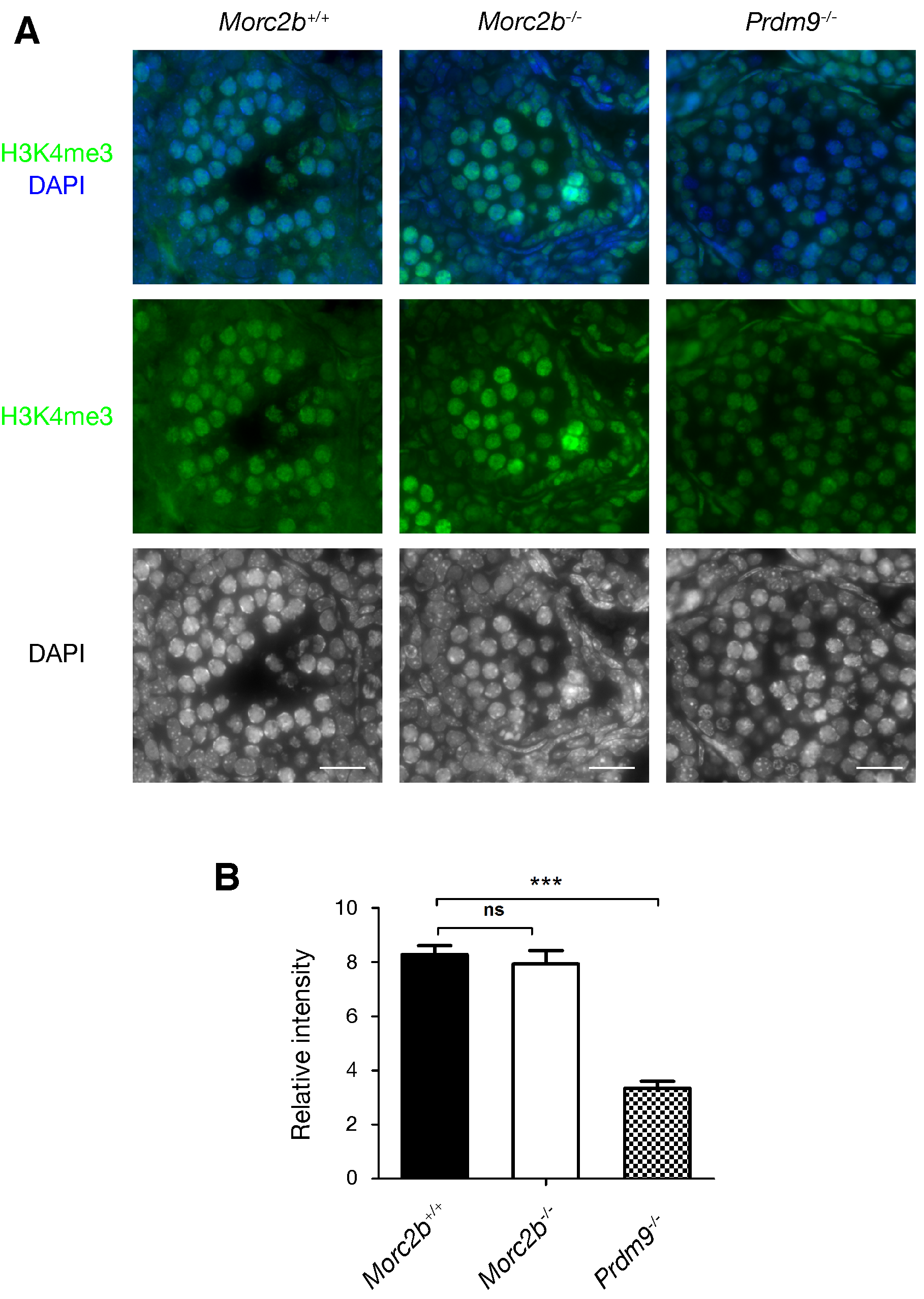

Supplement: S4 Fig — (A) Testis sections were immunostained with anti-H3K4me3 antibody. DNA was stained with DAPI. Scale bars, 25 μm. (B) Quantification of H3K4me3 fluorescence. The fluorescence in the most advanced spermatocytes (wild type, pachytene; mutant, pachytene-like) and Sertoli cells were quantified using Image J. The Y axis shows the relative intensity (spermatocyte/Sertoli cell). *, statistically significant (p < 0.05, Student’s t-test); ns, non-significant. (TIF) [file pgen.1007175.s004.tif]
