# Supplementary material for: MORC2B is essential for meiotic progression and fertility
Source: PLoS Genet. 2018 Jan 12;14(1):e1007175. doi: 10.1371/journal.pgen.1007175 (PMC5785033; doi:10.1371/journal.pgen.1007175)
Supplement: S2 Fig — Multiple sequence alignment was performed using CLUSTAL Omega (v1.2.1). Protein sequence accession numbers were as shown in S1 Fig. The GHKL-type ATPase and PHD zinc finger domains are highlighted. Identical and similar residues are indicated by asterisks and semicolons, respectively. (DOCX) [file pgen.1007175.s002.docx]

**S2 Figure. Alignment of Mouse MORC Proteins**

Multiple sequence alignment was performed using CLUSTAL Omega (v1.2.1). Protein sequence accession numbers were as shown in Figure S1. The GHKL-type ATPase and PHD zinc finger domains are highlighted. Identical and similar residues are indicated by asterisks and semicolons, respectively.

MORC1 -----------------------MDKYALLQRAKLHLDFIHANSTTHSFLFGALAELLDN 37

MORC2A ---------------------MAFTNYSSLNRAQLTFEYLHTNSTTHEFLFGALAELVDN 39

MORC2B ---------------------MAFTNYSTLNRAQLTFDYLHTNSTTHAFLFGALAELIDN 39

MORC3 ---------------------MAAQPPTGIRLSALCPKFLHTNSTSHTWPFSAVAELIDN 39

MORC4 MLLYRGAPAGPGTPGGGLARAGSVPQAFRIRLSTMSPRYLQSNSSSHTRPFSAIAELLDN 60

:. : : ::::**::* *.*:***:**

GHKL-type ATPase domain

MORC1 ARDAGAVRLDVFSVDNETLQGGFMLCFLDDGCGMSPDEASDVIYFGTSKKRLST-LKFIG 96

MORC2A ARDADATRIDIYAERREDLRGGFMLCFLDDGAGMDPSDAASVIQFGKSAKRTPE-STQIG 98

MORC2B ARDADATRIDIYAEKREDLQGGFMLCFLDNGVGMDPNDVINVIQFGKSAKRTPE-STQIG 98

MORC3 AYDPDVNAKQIWID-KTVISDHICLTFTDNGNGMTADKLHKMLSFGFSDKVTMNGHVPVG 98

MORC4 AVDPDVSARTVFID-VEEVKKKPCLTFTDDGCGMTPHKLHRMLSFGFTDKVIKKSQRPIG 119

* * . :: : * * *:* ** . :: ** : * :*

MORC1 QYGNGLKSGSMRIGKDCILFTKKEETMTCLFFSQTFCEKEGLTEVVVPIPSWLTRTRESI 156

MORC2A QYGNGLKSGSMRIGKDFILFTKKEDTMTCLFLSRTFHEEEGIDEVIVPLPTWNARTREPI 158

MORC2B RYGNGLKSGSMRIGKDFILFTKKENTMSCLFLSRTFHEEEGIDEVIVPLPTWNSQTREPV 158

MORC3 LYGNGFKSGSMRLGKDAMVFTKNGETMSVGFLSQTYLEVIKAEHVVVPIVTFNKHR-QMI 157

MORC4 VFGNGFKSGSMRLGKDALVFTKNGNTLAVGLLSQTYLECIQAQAVIVPIVPFSQQNKKMI 179

:***:******:*** ::***: :*:: ::*:*: * *:**: : : : :

MORC1 TDDPQKFFTELSIIFKYSPFKTEAELMQQFDMIYGRCGTLLIIYNLKLLLSGEPELDVTT 216

MORC2A TDNVEKFAIETELVYKYSPFHTEEQVMNQFMKIPGNSGTLVIIFNLKLMDNGEPELDIIS 218

MORC2B TDNMEKFAIETELIYKYSPFHTEEEVMTQFTKISGTSGTLVVIFNLKLTDNGEPELDVTS 218

MORC3 NLT--ESKASLAAILEHSLFSTEQKLLAELNAIMGKKGTRIIIWNLRSYKN-ATEFDFEK 214

MORC4 VTE--DSLPSLEAILNYSIFNCEKDLLSQFDAIPGKKGTRVLIWNIRRNKDGKSELDFDT 237

. . : ::* * * .:: :: * * ** ::*:*:: . *:*. .

MORC1 DKEDILMAEAPE--EI--------------P-ERRSFRAYTAVLYFEPRMKIFIQAKRVQ 259

MORC2A NPKDIQMAETSPEGTK--------------P-ERRSFRAYAAVLYIDPRMRIFIHGHKVQ 263

MORC2B NPKDIRMAEISQEGVK--------------P-ERHSFCAYAAVLYIDPRMRIFIHGHKVQ 263

MORC3 DKYDIRIPEDLDETAGRKGYKKQERMDQIAPESDYSLRAYCSILYLKPRMQIIIRGQKVK 274

MORC4 DQYDILVSDFDAEEKE------IGGVTSELPETEYSLRAFCSILYMKPRMKIFLRQKKVT 291

: ** : : * *: *: ::**:.***:*::: ::*

MORC1 TKHLCYSLYKPRKYQYTTSSFKGKFKTEVQKAEEAVKRAELLFKEVQ-----------AK 308

MORC2A TKRLSCCLYKPRMYKYTSSRFKTRAEQEVKKAEHVARIAEEKAREAESKARTLEVRMGGD 323

MORC2B TKKLCCCLYKPRKYTFTSHRFKTRAEQEVKKADQVAQLAEEKAREAESKARTLEIHMGGD 323

MORC3 TQLVSKSLAYIERDVY-------------------------------------------- 290

MORC4 TQMIAKSLANVEYDIY-------------------------------------------- 307

*: :. .* . :

MORC1 VNQPDRIALSSTQDALQKALQDVDTKHKSLRQKQRALRKARTLSLFFGVNTEDQHQAGMF 368

MORC2A LTRDSRVMLRQVQNTAITLRREADVKKRIKDAKQRALKEPKELNFVFGVNIEHRDLDGMF 383

MORC2B ITRDSRVMLRQVQNTAITLRREADVKKRIRDAKQQALKEPKELTFVFGVNIEHRDHDGMF 383

MORC3 ----------------------------------RPKFLTRTVRITFGFNCRNKDHYGIM 316

MORC4 ----------------------------------KPTSTNKQVRITFGFSCKYHNQFGVM 333

: : : : **.. . :. *::

MORC1 IYSNNRLIKMYEKVGPQLKMKSLLGAGIIGIVNIPLETMEPSHNKQEFLNVQEYNHLLKV 428

MORC2A IYNCSRLIKMYEKVGPQLEGG-MACGGVVGVVDVPYLVLEPTHNKQDFADAKEYRHLLRA 442

MORC2B IYNCSRLIKMYEKVGPQLEKG-MVCGGVVGVIDVPYLVLEPTHNKQDFADAKEYRHLLRA 442

MORC3 MYHKNRLIKAYEKVGCQLKAN-NMGVGVVGIIECNF--LKPTHNKQDFDYTNEYRLTILA 373

MORC4 MYHNNRLIKAFEKAGCQLKPTCGEGVGVIGVIECNF--LKPAYNKQDFEYTKEYRLTINA 391

:* .**** :**.* **: *::*::: ::*::***:* .:**. : .

MORC1 MGQYLIQYCKDIGISNRNLTLFWDEFKYQHSKDTDSSLESLQWRRRQAMGIPFILQCDLC 488

MORC2A MGEHLAQYWKDIAIAQRGIIKFWDEFGYLSANWNQPPSSELRFKRRRAMEIPTTIQCDLC 502

MORC2B MGEHLAQYWKDIEIAQHGIIKFWDEFGYLSANWNRPPSDELHFKRKRAMQVPTTIQCDLC 502

MORC3 LGEKLNDYWNEMKVKKN--------AEYP-------VN--LPVEDIQKRPDQTWVQCDAC 416

MORC4 LARKLNAYWKEKISQEN--------F--------------EPLPTSRRIPDQTWVQCDEC 429

:.. * * :: :. : :*** *

PHD zinc finger domain

MORC1 LKWRVLPSSSNYQEKGLPDLWICASNPNNLENSCNQIERLPSIPLGTVNRRPPSKDERER 548

MORC2A LKWRTLPFQLSSVETDYPDTWVCSMNPDPEQDRCEASEQKQKVPLGTLKKDPKTQEEKQK 562

MORC2B LKWRTLPFQLSAVEEGYPINWVCSMNPDPEQDQCEAFELKQKIPLGILKKAPKTQEERQK 562

MORC3 LKWRKLPDGID----QLPEKWYCSNNPDPQFRNCEVPEEPEDEDL--VHPTYEKTYKKT- 469

MORC4 LKWRRLPGMVD--PSTLPARWFCYYNPHPKFKRCSVPEEQERIDEDLHRSKAKQQVEAAE 487

**** ** . * * * **. *. * . :

MORC1 QLQESVQ-------RYQDKLVEAQPQ---KSQLIVTSKIP-EFKSSCLSSALKEKSKLG- 596

MORC2A QLTEKIR-------QQQEKLEALQKTTPIRSQADLK-KLPLEVTTRPIEEPV-RRPQRP- 612

MORC2B QLTEKIQ-------QEQRKLKALKKIKPIHSQSDLK-KLPLEVTSRPFSKYP-AHIFQG- 612

MORC3 ------SKERFRI-RQPEILPRI--LPQINPELLYQ----TSVSSQSFSP-VKE-SVPRP 514

MORC4 KKQKPMESDKYQVFSNPPKTPPLQDMAELNDKTIGY----EQINSPSLLPSVREESRSPP 543

. : ...: :

MORC1 -----RIQPSGA------DLT--QGSPS--------SVKLSFMQRSQKRSTEDTHSDVEF 635

MORC2A -----RSPPLPA------VIKNAPSRPP--------SIQTPR--------PSTQLRKTSV 645

MORC2B -----PQSSFHV------VKTNARRRPQ--------SRHA----------PFRQLQRSSI 643

MORC3 HLSEVTS-PFA-ARIINLNLASPASEPENSSMKRKLGVHSSILNAKTRR----------- 561

MORC4 RLKSLDSSAFQISRKYKLIL---------------------------------------- 563

MORC1 ICMTKIPKKSV----KKTVKYLQPGHAPALLENLKLED---------------------- 669

MORC2A I-SLPKPPTTAARGETSTSRLLQPTEAPRKPANPPIKT---------------------- 682

MORC2B ICTNPKPPFLVD---KTEAVLLQPPETPQKSVSLLVKT---------------------- 678

MORC3 LSNPPVENSSYKNDDDEDVIILEENSTPKPAVDLEVKSDIEVKSEQSHTEQSGIHVDLVS 621

MORC4 -GEEPVEKRRKIQ-----------TEMPLSPIDYSMSGFY------RRVEAATAYPEGEN 605

* . :.

MORC1 TAQV----------------SSREIKKQQ--------------SESLVQAGKA-STDVAS 698

MORC2A VPRPTPPVHTPPLSLIPSSKSLREVPAQKAIKTPVVKKPEPPVKQSVATSGRKRSLAVSD 742

MORC2B IPQPPPLVQSLSPSVVPKSNNPWKVETPQIMNTPVAEMPYVPVNPSLVICDHKRSPEVSD 738

MORC3 SPKPCVQASS----------------------------------TSTSTSR--------- 638

MORC4 SPDKCSSERSTPPHLIPEYPES---------------------NKHTEENR--------- 635

MORC1 SRD------PTVTMVWDQSSTKVSLKQE-------------------------------- 720

MORC2A EEEAEEEAEKRRERC---KRGKLAVKEEKKEA---------------------------- 771

MORC2B EIE---DEDRRKRMC---KRGRFTVKKEKIQA---------------------------- 764

MORC3 -------SDPGITVSTQTDAPGLTVKKE-----------------ESMEEDMGVRNGTAT 674

MORC4 -------EAPALCPGSQDQDQGFLLPEELEDQMPKLVAEESNRSSENIDKDMNK--G-PF 685

. . : :*

MORC1 ---------------E---EEEVPLIKPDK------------QELCDDTPVVKGNSSALH 750

MORC2A -------NELSDSAGE---DHPAELRKAQKDKGLHVEVRVNREWYTGRVTAVEVGKNAVR 821

MORC2B -------SELSDSSGE---ENPVDLKTAQKDKGLYVEVRMMGECYKGHVTAVEVGDNVVW 814

MORC3 LSCVGTEAKVQETSAESVDATSHQLQE--LRSELLVVTQ-ERDDYKRQCQMFTDQIQVLQ 731

MORC4 VAVVGVAKGVADSGA------PIQLVP--FNREEFVGKRKRAESWKRANP-YSSAAP--- 733

* :

MORC1 WKSLPGVQMEDLSPRSGH------------------------------------------ 768

MORC2A WKVKFDYVPTDTTPRDRWVEKGSEDVRLMKPPSPEHQSPD---TQQEGGEEEEAMVARQA 878

MORC2B WKVKFEDMPKDSTPRDCWVEKGSENVWLVKP-SPEYQSTD---EQQEDRKGEEDTVVQQA 870

MORC3 -QRLLEMNDKCVKKEKCHQSTETDAVFLLDSVNGQAESLDHLGSQYQQALQEIERLKRQC 790

MORC4 -------AATAGKGKDCQDSRSRNMPKIK-------------------TPKESEELKRTT 767

. ..

MORC1 -KINSVSGDCQLPASPMPSQSMSVEET---ARKLLSNLREILLYFVPEF-QLSS-EFECT 822

MORC2A VALPEPSTSDGLPIEPDTTATSPSHET---IDLLVQILRNCLRYFLPPSFPISKKELSVM 935

MORC2B LALQQTSTSECFCTEPDTTASTANHKT---IDLLVQILWNCLHYFMPLSFPISKKELGAM 927

MORC3 SALQQVKSECSQASCTES-K-SEVDEMAVQLDDVFRQLDKCTIER--DQYK---NEVQLL 843

MORC4 -----------------------------------EKLERVLAER--NLFQ---QKVEEL 787

* . :.

MORC1 SVEELITNPELERCPENINEKLKTCF-NQIQNIYMAQYEKRLKRKMQSIVY--------- 872

MORC2A NSEEL------------ISFPLKEYF-KQ--------YEVGLQNLCHSYQS--------- 965

MORC2B NSEEL------------LSLPLKECF-KQ--------YEVGLQNLCRSYQR--------- 957

MORC3 EIEKSHI---HSQCEE-LQ----------------------------------------- 858

MORC4 EQEKNHW---HSEYKK-AQHELVTYSTQETEGIYWSKKHMGYRQAEFQILKAELERTKEE 843

. *: .

MORC1 ---------EANRRGLL---NQVFL-GQCELKRKRTEEKLSDLRAKLALLLQKLQLGGPA 919

MORC2A ---------RADSRAKA---SEESL-RTSEKKLRETEEKLQKLRTNIVALLQKVQEDIDI 1012

MORC2B ---------CADSQAKV---SEESL-RISQKKLQETEEKLQKLRTNIQTLLQMAQQGINI 1004

MORC3 --TEVEQLKSTGQQAAA-DGSTASNAEEPVSYVDGESLKLRSLRVNVGQLLAMIVPDLDL 915

MORC4 KQELKEKLKETESHLEVLQKAQVSFRNPEGDDLERALARLTRLRVHVSYLLTSVLPHLEL 903

: : :* **..: **

MORC1 GD----PQQIDAYLEDLLKEDRLPTALHEKSPESA 950

MORC2A NT----DDELDAYIEDLITKGD------------- 1030

MORC2B RA----DDELDAYIEDLVSSDD------------- 1022

MORC3 QQVNYDVDVVDEILGQVVEQMSEISST-------- 942

MORC4 REIGYDSEQVDGILYTVLEANHILD---------- 928

: :* : ::
